# I See What You Mean: How Attentional Selection Is Shaped by Ascribing Intentions to Others

**DOI:** 10.1371/journal.pone.0045391

**Published:** 2012-09-26

**Authors:** Eva Wiese, Agnieszka Wykowska, Jan Zwickel, Hermann J. Müller

**Affiliations:** 1 Ludwig-Maximilians-Universität München (LMU Munich), Munich, Germany; 2 Birkbeck College, University of London, London, United Kingdom; CNRS - Université Claude Bernard Lyon 1, France

## Abstract

The ability to understand and predict others’ behavior is essential for successful interactions. When making predictions about what other humans will do, we treat them as intentional systems and adopt the *intentional stance*, i.e., refer to their mental states such as desires and intentions. In the present experiments, we investigated whether the *mere belief* that the observed agent is an intentional system influences basic social attention mechanisms. We presented pictures of a human and a robot face in a gaze cuing paradigm and manipulated the likelihood of adopting the intentional stance by instruction: in some conditions, participants were told that they were observing a human or a robot, in others, that they were observing a human-like mannequin or a robot whose eyes were controlled by a human. In conditions in which participants were made to believe they were observing human behavior (*intentional stance likely*) gaze cuing effects were significantly larger as compared to conditions when adopting the intentional stance was less likely. This effect was independent of whether a human or a robot face was presented. Therefore, we conclude that adopting the intentional stance when observing others’ behavior fundamentally influences basic mechanisms of social attention. The present results provide striking evidence that high-level cognitive processes, such as beliefs, modulate bottom-up mechanisms of attentional selection in a top-down manner.

## Introduction

Can we design machines that think? This old question has not yet been settled, despite the progress in the fields of artificial intelligence and cognitive science. For us humans, however, equally important questions are: would we be inclined to treat thinking artificial systems equally to other humans, and would we be ready to engage in social interactions with non-human agents that have minds? Alan Turing postulated that observed behavior is the only source of information based on which we ascribe minds to others [1]. Accordingly, for us to engage in social interactions, it would be critical that we *perceive* other agents as thinking, whether or not they *actually* have a mind.

In this paper, we ask a fundamental question, namely: would the *mere belief* that an agent has a mind be sufficient to engage in interactions with him/her in a social way, compared to if he/she was believed to be just a machine. The belief that an agent has a mind might lead us to adopt the *intentional stance* [2] towards him/her, which involves “treating the object whose behavior you want to predict as a rational *agent* with beliefs and desires and other mental states” (Dennett, 2003, p. 372). Dennett argues that the intentional stance is the best predictive strategy, given that the system whose behavior we want to predict is truly intentional. Throughout our lifelong experience with other humans, we have learned that humans are truly intentional systems in this sense – and, therefore, adopting the *intentional stance* towards human agents should be more successful in predicting their behavior as compared to adopting other strategies (e.g., the design or the physical stance).

Importantly, adopting the intentional stance towards other agents might not only be a successful predictive strategy, but also play a decisive role for one’s own readiness to engage in social interactions. For instance, if I believe that you are pointing to a location with the intention of showing me something, I will be likely to direct my attention there; but I will be unlikely to attend to where a lever of a broken machine is pointing, as I will not interpret the lever’s behavior as conveying communicative content [3], [4]. The present study was designed to investigate the fundamental issue of whether a belief concerning the minds of others has an impact on *basic* mechanisms underlying social cognition.

### Understanding Others’ Behavior in Social Interactions

On the neuronal level, interpreting others’ intentions is supported by a system consisting of *medial prefrontal cortex* (mPFC), *superior temporal sulcus* (STS), *orbitofrontal cortex* (OFC), *amygdala* and *anterior insula* [5], [6], [7]. Furthermore, activation in the *anterior paracingulate cortex* has been reported to be related specifically to the adoption of the intentional stance [8]. At the cognitive level of description, Baron-Cohen [9] has postulated that higher-level processes of mental-state attribution (*Theory of Mind*, ToM) are informed by low-level perceptual mechanisms: the *Intentionality Detector* (ID) and the *Eye-Direction Detector* (EDD).

Although the interpretation of social scenes involves low-level perceptual processes, it is unlikely that information is fed forward unidirectionally to higher-level processes of mental-state attribution, as everyday interactions require mechanisms that modulate perceptual information according to its social relevance. For these reasons, Teufel and colleagues [10], [11], [12] proposed that low-level processes not only inform higher-level processes but are themselves modulated by the latter, that is: specifying particular mental states requires the integration of bottom-up information provided by the stimulus and top-down information reflecting various context variables. In support of this view, Teufel and colleagues [11] reported that adaptation to gaze direction depended on whether participants believed that the observed person could actually or could not see through a pair of goggles.

### Reading Out Others’ Mental States Based on Gaze Direction

Gaze direction provides the basis for making inferences about the other’s focus of interest, and encourages the observer to shift attention to the corresponding location [13]. Attention shifts triggered by gaze direction are typically investigated using a gaze-cuing paradigm [14], in which a schematic face is presented centrally on the screen that gazes either straight ahead or to the left or right. Targets appearing in the gaze-cued hemifield are detected, localized, and discriminated more rapidly compared to targets in the opposite, uncued hemifield.

Orienting attention in response to perceived gaze direction has traditionally been regarded as a reflexive, bottom-up process involved in making inferences about the other’s mental states [15]. In line with this view, children as young as three months have been found to attend more quickly to peripheral objects that are gazed at by a human face compared to objects not directly gazed at [16]. Moreover, adult participants have been found to reflexively orient attention in the direction of another’s gaze even when gaze cues are counterpredictive of the target location [17], whereas they voluntarily orient away from counterpredictive arrows [18] or extended tongues [19].

However, although gaze-cuing has been shown to be triggered in a bottom-up fashion, a growing body of evidence suggests that attending to where others gaze is not purely reflexive, but can rather be modulated by higher-level cognitive processes. For instance, Ristic and Kingstone [20] presented participants with an ambiguous stimulus that could be perceived as either a car with wheels or a face with a hat, and manipulated their beliefs by instruction; the stimulus was found to cue participants’ attention only when they believed that they were looking at a face, rather than at a car. Similarly, Kawai [21] found that schematic faces caused gaze-cuing effects only when participants believed that a potential target was visible to the gazer, but not when it was occluded.

### Aim of Study

The present study was designed to address a more fundamental issue, namely, whether adopting the intentional stance based on a mere belief concerning the observed agent would affect basic mechanisms of social attention. In classical studies examining the processes involved in inferring others’ mental states [22], [23], participants are typically observing *intentional* agents and asked to provide a description of the agents’ behavior making use of mentalistic vocabulary. Similarly, in the studies of Teufel and colleagues [11], [12], participants were always observing intentional agents exhibiting particular mental states (such as being either able or unable to see through a pair of goggles). However, inferring particular mental states from observed behavior presupposes that the agent is construed as an intentional system that is *capable* of having intentions. Given this, based on previous studies, one cannot decide whether basic mechanisms of social perception and attention were influenced by adopting the intentional stance *per se*, rather than by processes of reasoning about *particular* mental states. For resolving this fundamental issue, it is important to examine whether the likelihood of adopting the intentional stance *per se* (i.e., assuming that the observed agent has mental states) has an effect on one’s own social cognition.

Thus, critically, we distinguish between processes of *mentalizing* about others’ internal states and *adopting the intentional stance*. The former involves an active process of reasoning about mental states that underlie particular behavior, whereas the latter is, fundamentally, a result of activating a set of pre-existing representations of what it means to be “a human”, which contains – amongst others – properties like “having a mind” or “being capable of having intentions. By contrast, a representation of a mechanistic device (such as a robot) is probably devoid of mind-related characteristics. Thus, when predicting and/or explaining behavior of observed systems, humans either adopt the intentional stance or use other predictive strategies (such as the design or physical stances), dependent on the activated representations. In sum, adopting the intentional stance is based on a decision as to whether or not an observed agent is capable of having intentions; mentalizing, by contrast, is concerned with reasoning about what specific intentions are underlying behavior displayed by an agent that has already been classified as having a mind.

### Experiments

**Figure 1:**
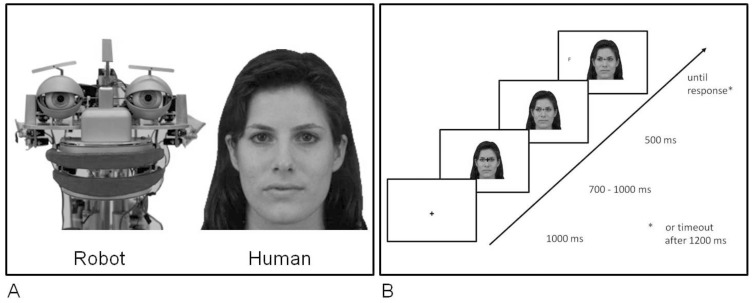
Stimuli and trial sequence. Pictures for the Robot and Human Gazer are shown in (A). The sequence of events within a trial is shown in (B). The human face (F 07) is taken from the Karolinska Directed Emotional Faces (KDEF) database [24]. We have received written informed consent (as outlined in the PLoS consent form) from Karolinska Institute (Department of Clinical Neuroscience, Section Psychology) to use the photograph for experimental investigations and illustration of the stimuli in publications. The picture of the robot face is made by LSR (TU Munich) and depicts the research robot EDDIE (made by LSR, TU Munich).

In three experiments, we investigated whether social attention is modulated by the likelihood of adopting the intentional stance. To this end, we used two different types of gazers to orient participants’ attention in a gaze-cuing paradigm: either a human face or a robot face (see *Figure 1A*). The likelihood of adopting the intentional stance was manipulated by instruction rather than by the appearance of the gazer (see [8]). In **Experiment 1**, participants were instructed (*Instruction 1*) that they would observe either a human (*intentional stance likely*) or a robot (*intentional stance unlikely*). In **Experiment 2**, *Instruction 2* informed participants that they were observing a human or a robot whose gaze behavior was controlled by a human (*intentional stance likely* in both cases), while in *Instruction 3* participants were told they would be observing a human-like mannequin or a robot (*intentional stance unlikely* in either case). In both experiments, participants had to perform a target discrimination task. To ensure that variations of gaze-cuing effects could not be attributed to physical differences between stimuli, the same stimuli were used for all experimental groups. On the intentional-stance hypothesis, we expected to find stronger gaze-cuing effects for stimuli representing *intentional agents* (Experiment. 1: *human*; Experiment 2, Instruction 2: *human* and *robot*) relative to stimuli representing agents who were less likely to be treated as intentional systems (Experiment 1: *robot*; Experiment 2, Instruction 3: *human* and *robot*). Please note that our paradigm did not involve actual social interaction. That is, participants observed only photographs representing either intentional or non-intentional agents. Yet, we believe that it is still possible to adopt the intentional stance towards virtual humans. For example, when one watches a movie, one predicts behavior of characters depicted in that movie by adopting the intentional stance, even though one is not actually interacting with the characters. At the same time one might not adopt the intentional stance to a virtual robot due to pre-existing representation of what a robot is **Experiment 3** examined whether the effects in question are generalizable to tasks with different attentional demands. 1. The pattern of results revealed in Experiments 1 and 2 was replicated in Experiment 3 (localization task), which is reported in the supporting information section (*Text S1*). Please see also *Figure S1* and *Table S1*.

## Materials and Methods

### Participants

24 participants participated in ***Experiment 1*** (15 women; mean age: 25 years (M = 25.25, range: 19–32), two left-handed). In ***Experiment 2***, 48 participants were randomly assigned to two groups with different instructions: *Instruction 2* (human – human controlled robot): 16 women; mean age: 23 years (M = 23.17, range: 18–30) and *Instruction 3* (human-like mannequin, robot): 18 women; mean age: 23 years (M = 22.58, range: 18–30). Participants reported normal or corrected-to-normal visual acuity. Testing time was about 40 minutes. One participant had to be excluded from Experiment 1 because of significantly increased error rates compared to other participants (M = 16.7% compared to M = 4.7%); one participant was excluded from Experiment 2 (Instruction 2) because the participant did not complete the experiment.

### Ethics Statement

The experiments were conducted at the Department of Experimental Psychology at the LMU Munich, where all experimental procedures with purely behavioral data collection (e.g., RTs and error rates) of healthy adult participants, that do not include invasive or potentially dangerous methods are approved by the ethics committee of the Department of Psychology, LMU Munich, in accordance with the Code of Ethics of the World Medical Association (Declaration of Helsinki). Data were stored and analyzed anonymously. Participants gave their informed consent and were either paid or received course credit for participating.

### Apparatus

Stimuli were presented on a 17-in. Graphics Series G90fB monitor with the refresh rate set at 85 Hz. RT measures were based on standard keyboard responses. Participants were seated approximately 57 cm from the monitor, and the experimenter ensured that participants were centered with respect to the monitor. The experiment was controlled by *Experiment Builder* (SR Research Ltd., Ontario, Canada).

### Stimuli

In the human condition, digitized photos of a female face (F 07) were used as stimuli, chosen from the *Karolinska Directed Emotional Faces* database [24]. We have received written informed consent (as outlined in the PloS consent form) from Karolinska Institute (Department of Clinical Neuroscience, Section Psychology) to use the photograph (F 07) for experimental investigation and illustration of the stimuli in publications. In the robot condition, photos of a humanoid robot (*EDDIE; developed by TU Munich*) were used. Stimuli were 6.4° wide and 10.0° high, depicted on a white background and presented in full frontal orientation with eyes positioned on the central horizontal axis of the screen (*Figure 1A*). For left- and rightward gaze, irises and pupils in the eyes were shifted with Photoshop_TM_ and deviated 0.4° from direct gaze. The target stimulus was a black capital letter (F or T), measuring 0.8° in width and 1.3° in height. Targets appeared on the horizontal axis, located 6.0° from the center of the screen.

### Procedure

*Figure 1B* illustrates the sequence of events in the present experiments. The beginning of every trial was signaled by a fixation cross at the center of the screen. 1000 ms later, a face with straight gaze appeared on the screen while the fixation cross remained in its position. After a random time interval of 700 to 1000 ms, gaze either remained straight or was shifted left- or rightwards. Following the gaze cue with a SOA of 500 ms, the target letter appeared either on the left or the right side of the screen. SOA was measured as the interval between the onset of the gaze shift to the onset of the target. Face and target remained on the screen until a response was given or after 1200 ms had elapsed. The inter-trial-interval (ITI) was 680 ms.

At the beginning of each session, participants were told to fix their gaze on a centrally presented cross. They were also instructed that after the fixation cross a photo of either a human or a robot would appear in the center of the screen but that they should still keep their eyes fixated on the fixation cross. Further, participants were advised that after its initial presentation the face gaze could remain straight or shifted left- or rightwards, subsequently followed by a target letter. Participants were asked to respond to target identity as quickly and as accurately as possible. For half of the participants F was assigned to the “D” key and T to the “K” key on the keyboard, for the other half of the participants stimulus-response mapping was reversed. The key labels were covered with a sticker to prevent letter interference effects. All instructions were given in a written form and the experimenter was not informed about the purposes of this experiment.

Each session of the experiment was composed of 500 trials, with a block of 20 practice trials preceding 10 experimental blocks of 48 trials each. Gaze direction (straight, left, right), target side (left, right), target identity (F, T) and cue identity (human, robot) were selected pseudo-randomly and every combination appeared with equal frequency. Gaze direction was manipulated orthogonal to target position: that is, in one third of the trials, gaze was directed to the side on which the target appeared (*valid*), in another third of the trials to the other side (*invalid*) and in another third of the trials, the face was gazing straight ahead (*neutral*).

### Analysis

Gaze-cuing effects were examined by comparing valid vs. invalid trials, i.e., in terms of costs-plus-benefits (invalid-valid) rather than benefits (neutral-valid) and costs (invalid-neutral) with respect to the neutral condition. This was done because the latter condition may not provide an adequate baseline measure for the separate assessment of cuing effects [25]. In fact, in all conditions of the present study, neutral trials were found to elicit longer RTs than valid and invalid trials – for two likely reasons: (i) straight-ahead gaze might have a holding effect on attention [26], making it difficult for the target onset to disengage attention and summon an orienting response (in line with [27]); (ii) with straight-ahead gaze being maintained in the neutral condition, there was no similar temporal warning-signal effect to that induced by the gaze shift in valid and invalid conditions. In this regard, gaze cuing paradigms with naturalistic faces differ from those with schematic faces [14]: in the latter, trials typically start with face-like stimuli in which the eyes contain no pupils; pupils appear only later, so that also straight-ahead gaze involves a visual change that can serve as a temporal warning signal. This is not done with naturalistic faces [28], as empty eyes without pupils are thought to be emotionally disturbing, potentially interfering with attentional orienting.

## Results

### Experiment 1

**Figure 2:**
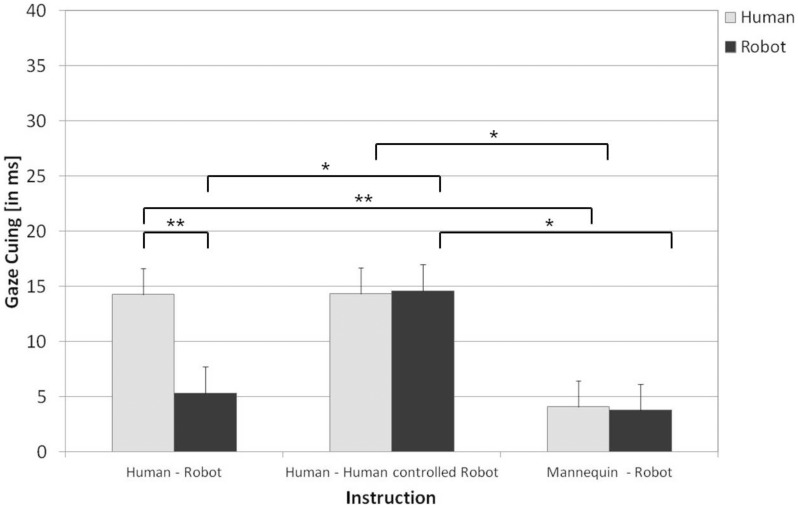
Size of gaze cuing effects as function of Cue Type and Instruction. Error bars represent standard errors of the mean adjusted to within-subject designs (see [40]). *p<.05, **p<.01.

Missed (0.69%), and incorrect responses (3.69%), as well as RTs deviating by more than ±2.5 SD from individual participants’ means were removed prior to analyses. The statistical analyses focused on the conditions of interest: mean RTs on valid versus invalid trials as a function of cue type (human vs. robot). Results of statistical analyses for all trial types (neutral, valid, invalid) are presented in *Table 1*, along with Mean RTs and Standard Errors. *Figure 2* depicts the corresponding gaze-cuing effects (Δ_RT_invalid-valid) for both types of cue; for the results of Experiment 1, see the top row in *Table 1* and the left-hand side of *Figure 2*.

A two-way repeated-measures analysis of variance (ANOVA) of mean RTs with the factors cue validity (valid, invalid) and cue-type (human, robot) revealed the main effect of validity [*F*(1,22) = 22.131, *p*<.001, *η*_p_^2^ = .501] to be significant: valid trials yielded faster responses than invalid trials (449 ms vs. 458 ms). Importantly, the interaction between validity and cue-type was significant [*F*(1,22) = 14.113, *p*<.002, *η*_p_^2^ = .391]: under ***Instruction 1***, gaze-cuing effects were twice as strong for the human (Δ_RT_ = *14 ms*, [*t*(22) = -5.954, *p*<.001]) as for the robot (Δ_RT_ = *5 ms*, [*t*(22) = -2.211, *p*<.04]), though reliable in both conditions. The main effect of cue-type (454 ms for the human vs. 453 ms for the robot) was not significant [*F*(1,22) = .017 *p*>.8, *η*_p_^2^ = .001].

### Experiment 2

Experiment 2 was designed to further investigate the influence of adopting the intentional stance on gaze-cuing, independently of the physical characteristics of the stimuli. To realize this, stimuli were kept the same across conditions, while instruction was manipulated: ***Instruction 2***– human versus robot controlled by human (*intentional stance likely*) and ***Instruction 3***– human-like mannequin versus robot (*intentional stance unlikely*). The setup was comparable to Experiment 1, with one exception: in order to investigate the temporal dynamics of attentional orienting in response to gaze cues, the stimulus onset asynchrony (SOA) between cue and target presentation was varied: SOA was either short (250 ms) or long (600 ms).

Missed (0.44%) and incorrect responses (4.20%) as well as outliers (±2.5 SD from individual participants’ means) were excluded from analysis. For the results of Experiment 2, see the middle (Instruction 2) and bottom (Instruction 3) rows in *Table 1* and the middle (Instruction 2) and right-hand sides (Instruction 3) of *Figure 2*. Mean RTs were examined in a mixed-design ANOVA with the between-subject factor instruction (Instruction 2, Instruction 3) and the within-subjects factors SOA (250 ms, 600 ms), validity (valid, invalid), and cue-type (human, robot). Results of statistical analyses for all trial types (neutral, valid, invalid) are summarized in *Table 1*. As SOA did not interact with any effects of interest, all *F*s <1.3, *p*s >.2, data were collapsed over this factor.

**Table 1:** Mean RTs and SEM (in ms) as a function of cue validity and instruction, for human and robot cues.

	Human			Robot			Statistics
	Valid	Invalid	Neutral	Valid	Invalid	Neutral	Cue type×Validity
Instruction 1	447 (10)	461 (11)	479 (10)	451 (11)	456 (12)	481 (11)	*F*(2,44) = 3.7, *p*<.05
Instruction 2	454 (12)	469 (13)	482 (12)	453 (12)	467 (12)	484 (12)	*F*(2,44) = 0.1, *p*>0.8
Instruction 3	451 (11)	455 (12)	474 (12)	450 (11)	454 (11)	477 (13)	*F*(2,46) = 0.4, *p*>0.6

Again, there was a main effect of validity [*F*(1,45) = 33.790, *p*<.001, *η*_p_^2^ = .429], with shorter RTs for valid relative to invalid trials (452 ms vs. 461 ms), and no main effect of cue type (457 ms for the human vs. 456 ms for the robot, [F(1,45) = .641, *p*>.4, *η*_p_^2^ = .014]). Most importantly, the interaction between validity and instruction was significant [*F*(1,45) = 11.087, *p*<.003, *η*_p_^2^ = .02]: gaze-cuing effects were larger when adopting the intentional stance was likely (Δ_RT_ = *14 ms* for the human and Δ_RT_ = *15 ms* for the robot) compared to when this was unlikely (Δ_RT_ = *4 ms* for the human and for the robot). Note that this effect was independent of cue-type, as evidenced by a non-significant interaction between instruction, validity, and cue-type [*F*(1,45) = .012, *p*>.9, *η*_p_^2^<.001]. No other effect reached significance [*F*<.02, *p*>.8].

### Comparisons Across Experiments

To compare gaze-cuing effects among all three instructions and cue-types, post-hoc t-tests (Bonferroni-corrected for multiple comparisons) were conducted. Comparisons confirmed that the size of the gaze-cuing effect was not influenced by the cue-type as such (human vs. robot), but only by the likelihood of adopting the intentional stance towards the cue provider. In more detail, gaze-cuing effects did not differ between human and robot in conditions in which participants believed they observed either human behavior (Experiment 1, ***human***: Δ_RT_ = *14 ms*, vs. Experiment 2, Instruction 2, ***robot***: Δ_RT_ = *15 ms;* [*t*(44) = -.094, *p*>.9]) or non-human behavior (Experiment 1, ***robot***: Δ_RT_ = *5 ms*, vs. Experiment 2, Instruction 3, ***human***: Δ_RT_ = *4 ms;* [*t*(45) = .328, *p*>.7]). But the same cue-type elicited cuing effects of *different* sizes depending on whether or not the intentional stance was likely to be adopted towards the cue provider (Experiment 1, ***human***: Δ_RT_ = *14 ms*, vs. Experiment 2, Instruction 3, ***human:*** Δ_RT_ = *4 ms* [*t*(45) = 2.727, *p<*.01]; Experiment 2, Instruction 2, ***robot*** *:* Δ_RT_ = *15 ms*, vs. Experiment 1, ***robot*** *:* Δ_RT_ = *5 ms;* [*t*(44) = 2.644, *p<*.02]).

## Discussion

The present study investigated whether the *mere belief* that the observed stimulus is representing an agent with a mind influences basic social attention mechanisms, as measured by gaze-cuing effects. Rather than solely manipulating perceptual aspects of the cue provider [28]–[30], we varied participants’ beliefs about the gazer through instruction (while keeping the stimuli constant). We hypothesized that gaze-cuing would be increased when adopting the intentional stance was likely, whatever the identity of the gazer.

Our findings clearly support this hypothesis: while both human and robot induced attention shifts to gazed-at positions, cuing effects were twice as large when adopting the intentional stance towards the gazer was likely, as compared to when this was unlikely. In particular, gaze-cuing effects were significantly smaller for the robot than for the human when no explicit instruction was provided. Importantly, however, the same stimuli elicited gaze-cuing effects to varying degrees when different beliefs were induced: the human face condition yielded reduced cuing effects (comparable to the robot condition) when it was believed to represent a mannequin, while the robot face elicited enhanced cuing effects (comparable to the human condition) when it was believed to be controlled by a human. The results of Experiment 3 show that this pattern is robust, generalizing to other tasks with very different attentional demands, such as target localization [31].

This pattern of results shows that basic social attention mechanisms are modulated by the observers’ beliefs, induced solely by instruction, about whether or not the cue provider represents an intentional system. That is, social attention mechanisms are modulated fundamentally by the observer adopting the intentional stance towards others, rather than simply by attributing particular mental states [10]–[12], [21].

### Reflexive Behavior under the Control of Beliefs

The present results provide evidence that attentional orienting in response to gaze direction is not purely reflexive, but prone to top-down modulation induced by higher-level cognitive processes. That is, gaze direction triggers social attention mechanisms based on a combination of two components: a bottom-up component that reflexively directs attention to where others are looking, and a top-down component that incorporates social context information relating to the observed scene into attentional guidance. In the present study, the bottom-up component produced a weak attentional bias towards stimuli at the gazed-at location, whether or not participants construed the cue provider as an intentional agent (in line with [29], [30]). The top-down component came into play in conditions in which adopting the intentional stance towards the cue provider was likely. Thus, attentional mechanisms involved in low-level processes of social perception not only influence, but *are* themselves influenced by beliefs humans hold about social stimuli they observe (in line with [10]–[12]). Interactions between lower- and higher-level processes are also supported by neuroimaging evidence: while the STS appears to trigger bottom-up responses to social signals, top-down control of these responses is thought to originate from the mPFC, adapting the system to the social context of the scene [32]–[34].

In this context, it is important to distinguish between i) reflexive vs. top-down modulated shifts of attention on the side of the observer; and ii) reflexive vs. intentional shifts of gaze on the side of the gazer. As discussed, the present data show that attentional shifts on the side of the observer are due to a reflexive mechanism that can be modulated by top-down component. At the same time, one needs to note that the gaze shifts on the side of the gazer can also be either reflexive (due to attentional orienting to a salient event in the periphery) or intentional, i.e., carrying social communicative content (the gazer shifts his/her gaze to the periphery in order to communicate a certain intention to the observer). The manipulation in the present study was concerned rather with the latter, as there was no salient event in the periphery that the gazer could reflexively shift gaze to. Therefore, we discuss the results in the context of intentional gaze behavior on the side of the gazer.

### Humans do not Engage in Social Interactions with Just Any Agent

The finding that social attention processes are modulated by adopting the intentional stance when observing others’ behavior raises a fundamental question: why does the belief that another agent is an intentional system influence the way we allocate attentional resources? Clearly, an attentional system that is sensitive to social context information is highly advantageous from an evolutionary perspective: it permits adaptation to the social relevance of the scenario in which an interaction takes place. Allocating attention to where another person is attending serves the purpose of establishing *shared intentionality* [4], which enables us to engage in collaborative activities by sharing goals, intentions, knowledge, and beliefs with others. The present results suggest that humans opt to engage in shared intentionality only with those who are believed to have intentions and are expected to display predictable, goal-oriented behavior. Given this, humans might be reluctant to adopt the intentional stance when observing a robot as compared to other humans. Importantly, what is crucial for adopting the intentional stance and, as a result, for readiness to engage in social interaction is not whether the observed agent *actually* has mental states, but whether the agent is *believed* to have mental states.

Interestingly in this context, there have been several reports [35]–[39] that humans tend to provide mentalistic descriptions of the behavior of simple geometrical figures in dynamic motion scenarios. However, participants in those studies may not have actually adopted the intentional stance towards the observed stimuli, but only described behavior using mentalistic vocabulary – similarly to when one says “my computer did not want to start”. Alternatively, participants may have adopted the intentional stance by treating the geometric figures as *representations* of intentional agents – in a similar way to the present study where the robot was instructed to be controlled by a human. Hence, the novelty of our study is that through instruction manipulation, we triggered the activation of preexisting representations of the observed agents: the representation of a human as being an intentional agent, versus that of a robot being a mechanistic, non-intentional object. These representations in turn modulated the degree to which social attention mechanisms were employed.

Consequently, if humans tend not to adopt the intentional stance towards robots, they would ascribe less *social* relevance to its behavior compared to that displayed by humans. Hence, the present findings are not only of theoretical interest, but are also of significance to applied domains in which artificial systems are to be involved in interactions with humans (e.g., social robotics). If attribution of mental states is a crucial factor for enabling efficient social interactions, social robotics might need to address the issue of humans being hesitant to adopt the intentional stance towards a robot.

### Concluding Remarks

The present findings indicate that a *mere belief* that the observed agent represents a human triggers the concept of an *intentional agent*, and encourages adopting the intentional stance – in contrast to when the observed agent is believed to represent a mechanistic system (e.g., a robot). Consequently, social attention mechanisms are more readily employed when the intentional stance is adopted. This seems plausible, especially given that two types of intentions are communicated through gaze behavior that leads to directing others’ attention [4]: *referential* – what is the object of attention; and *social* – why do I direct your attention to this object? If an observer believes that the latter component is missing and is not convinced that the observed agent is capable of communicating social intentions, he/she might allocate attention to a lesser degree to the gazed-at object. On this basis, we propose that adopting the intentional stance plays a pivotal role in basic attention mechanisms involved in social interactions. For us humans to recruit these mechanisms, it seems not to matter whether the observed agents can *actually* think – but rather whether we *believe* they do!

## Supporting Information

Figure S1 **Size of gaze-cuing effects as function of Cue Type and Instruction.** Error bars represent standard errors of the mean adjusted to within-subject designs (see [40]). *p<.05, **p<.01.
(TIFF)

Table S1 **Mean RTs and SEM (in ms) as a function of cue validity and instruction, for human and robot cues.**
(TIFF)

Text S1 **Supporting Methods.**
(DOC)
