# Supplementary figures and images for: I See What You Mean: How Attentional Selection Is Shaped by Ascribing Intentions to Others
Source: PLoS One. 2012 Sep 26;7(9):e45391. doi: 10.1371/journal.pone.0045391 (PMC3458834; doi:10.1371/journal.pone.0045391)

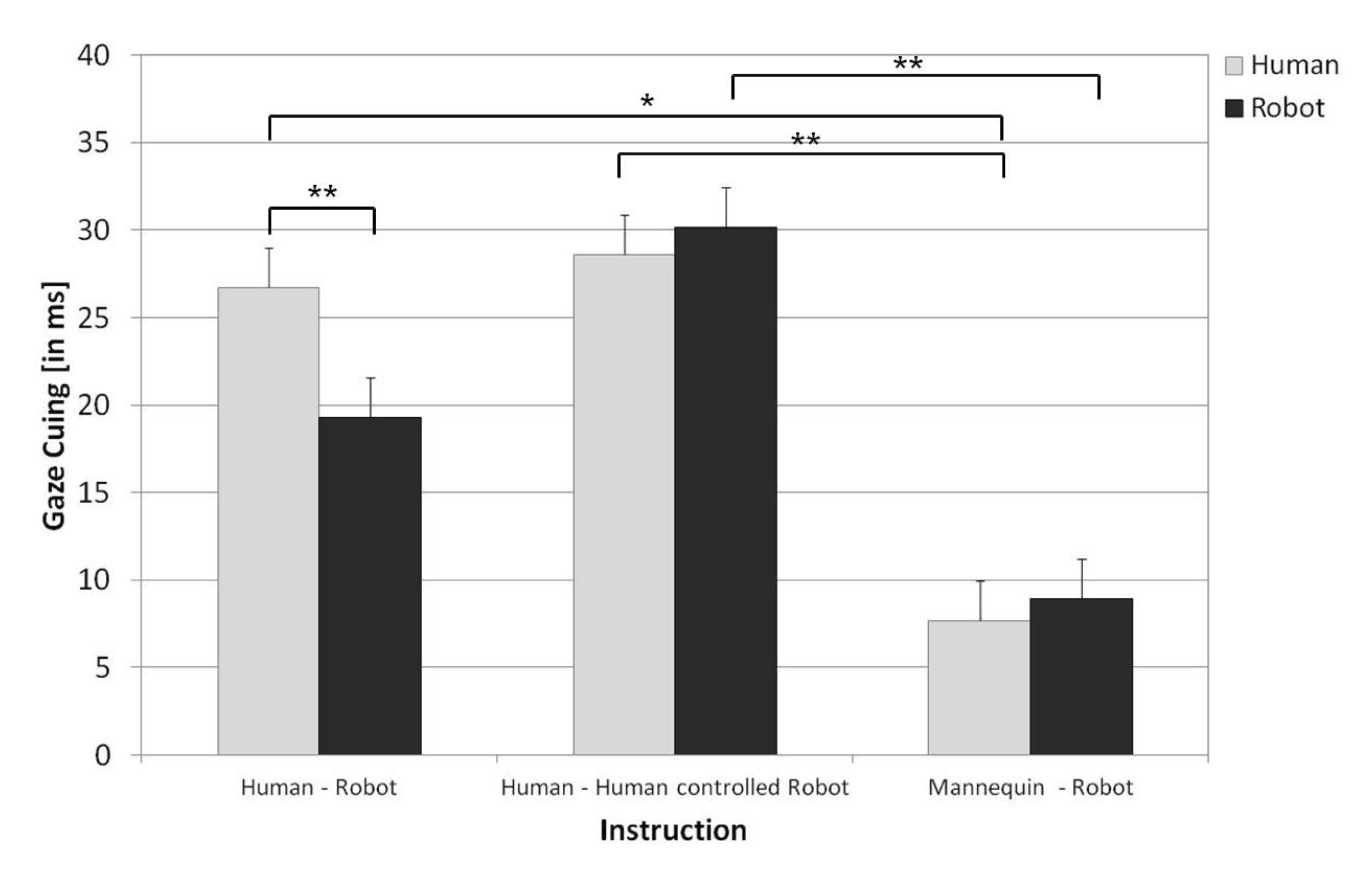

Supplement: Figure S1 — Size of gaze-cuing effects as function of Cue Type and Instruction. Error bars represent standard errors of the mean adjusted to within-subject designs (see [40]). *p<.05, **p<.01. (TIFF) [file pone.0045391.s001.tiff]

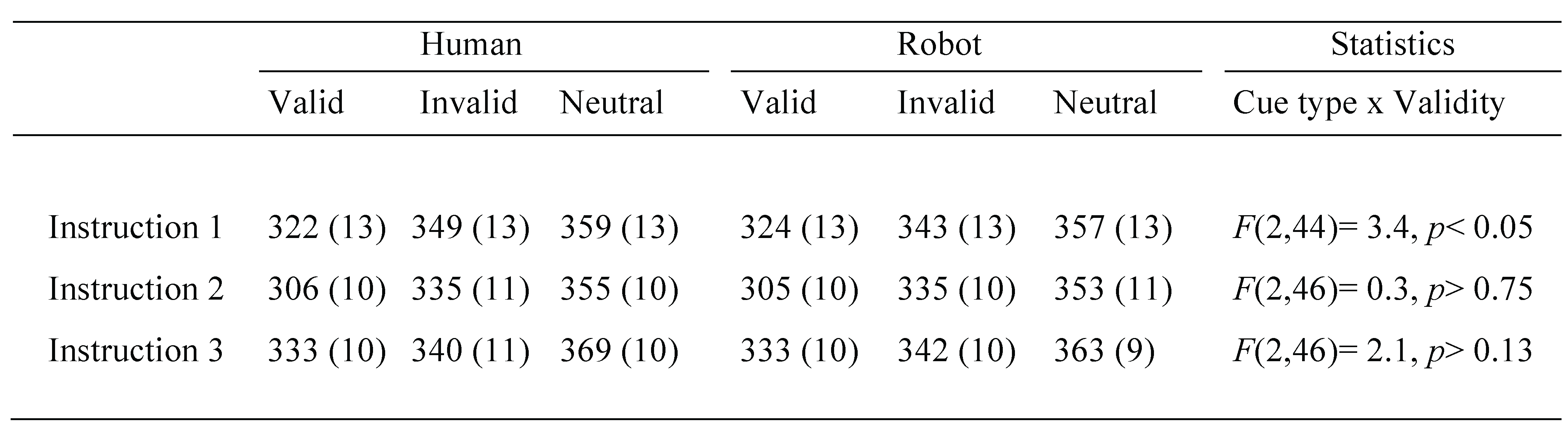

Supplement: Table S1 — Mean RTs and SEM (in ms) as a function of cue validity and instruction, for human and robot cues. (TIFF) [file pone.0045391.s002.tiff]
