# Supplementary material for: I See What You Mean: How Attentional Selection Is Shaped by Ascribing Intentions to Others
Source: PLoS One. 2012 Sep 26;7(9):e45391. doi: 10.1371/journal.pone.0045391 (PMC3458834; doi:10.1371/journal.pone.0045391)
Supplement: Text S1 — Supporting Methods. (DOC) [file pone.0045391.s003.doc]

**Supporting Information**

**Experiment 3**

Experiment 3 was conducted to examine whether the pattern of results revealed in Experiments 1 and 2 (discrimination task) would generalize to other types of task that make different demands on attentional processing resources. To this end, we selected a speeded target localization task, as this type of task is typically used in gaze cuing studies [14] and known to be sensitive to picking up small cuing effects.

**Method and Materials**

Experiment 3 was methodologically the same as Experiments 1 and 2 (instruction was varied between participants, while SOA, cue type, and cue validity were varied within participants), except for the task participants had to perform: participants were asked to localize the target as fast as possible by pressing the “D” key for targets presented on the left side and the “K” key for targets on the right side.

*Participants, Apparatus, Stimuli, Design and Procedure*

72 volunteers participated in Experiment 3, they were randomly assigned to three experimental groups with 24 participants each: ***Group 1*** (20 women; mean age: 25 years (M=25.00, range: 19-31); two left-handed) received *Instruction 1* (human, robot); ***Group 2*** (19 women; mean age: 25 years (M=24.79, range: 21-36); two left-handed) received *Instruction 2* (human, human-controlled robot); and ***Group 3*** (16 women; mean age: 25 years (M=24.71, range: 20-32); two left-handed) received *Instruction 3* (human-like mannequin, robot). None of the participants has taken part in either Experiment 1 or Experiment 2. One participant from Group 1 (Instruction 1) was excluded from analysis because of significantly increased error rates compared to other participants (M= 11.46% compared to M=1.38%).
*Ethics Statement*

The experiments were conducted at the Department of Experimental Psychology at the LMU Munich, where all experimental procedures with purely behavioral data collection (e.g., RTs and error rates) of healthy adult participants, that do not include invasive or potentially dangerous methods are approved by the ethics committee of the Department of Psychology, LMU Munich, in accordance with the Code of Ethics of the World Medical Association (Declaration of Helsinki). Data were stored and analyzed anonymously. Participants gave their informed consent and were either paid or received course credit for participating.

**Results**

Missed responses (0.34%), incorrect responses (1.04%), and outliers (±2.5 SD from individual participants’ means) were excluded from analysis. Mean RTs and Standard Errors for neutral, valid, and invalid trials are presented in *Table S1* as a function of validity, cue type, and instruction. *Figure S1* depicts gaze cuing effects as a function of cue type and instruction. As SOA did not interact with validity [*F*(1,68)= .197, *p*> .6, *η*_p_^2^= .003], and did not have an influence on the interaction between instruction and validity [*F*(2,68)= .058, *p*> .9, *η*_p_^2^= .002] or the interaction between validity, cue type and instruction [*F*(2,68)= .304, *p*> .7, *η*_p_^2^= .009], data were collapsed over this factor for subsequent analyses.

As can be seen from *Figure S1* (and *Table S1*), Experiment 3 closely replicated the results of Experiments 1 and 2. In more detail, valid cues yielded overall faster mean RTs than invalid cues [*F*(1,68)= 67.471, *p*< .001, *η*_p_^2^= .498]. When no further instruction was given (***Instruction 1***), the robot condition elicited smaller cuing effects than the human condition [*F*(1,22)= 9.821, *p*< .006, *η*_p_^2^= .309]. However, when both were believed to represent human behavior (***Instruction 2***), gaze cuing effects for the human and robot condition were of similar magnitude [*F*(1,23)= .296, *p*> .5, *η*_p_^2^= .013] and comparable to that in the human condition with no further instruction [*t*(45)= -.478, *p*= .635, *two-tailed*]. Similarly, when both agents were believed to represent non-human behavior (***Instruction 3***), the gaze cuing effects were also comparable in size [*F*(1,23)= .076, *p*> .7, *η*_p_^2^= .003] and even smaller compared to the robot condition with no further instruction [*t*(45)= 2.271, *p*< .03, *two-tailed*]. This overall pattern of results mirrors the findings of Experiments 1 and 2, showing that the essential effects are independent of the type of task.
